# Does altitude have an effect on stroke mortality and hospitalization risk? A comprehensive evaluation of United States data

**DOI:** 10.3389/fstro.2023.1223255

**Published:** 2023-10-03

**Authors:** Jacob Gerken, Nathan Huber, Demi Zapata, Ileana G. Barron, Isain Zapata

**Affiliations:** ^1^Department of Biomedical Sciences, Rocky Vista University, Parker, CO, United States; ^2^Department of Epidemiology, University of Alabama Birmingham School of Public Health, Birmingham, AL, United States

**Keywords:** sociodemographic, altitude, elevation, smoking, cardiovascular

## Abstract

**Background:**

Globally, stroke is a leading cause of death among people over 50 years of age. In the United States alone, over 150,000 people die due to stroke every year. Environmental factors such as altitude may play a role in these outcomes; however, their effects have not yet been comprehensively explored. The objective of this study is to evaluate the effect of altitude along with other covariates on stroke mortality and hospitalization rates in adults.

**Methods:**

This study utilized altitude, stroke mortality and hospitalization rates; antihypertensive and cholesterol-lowering medication usage; smoking prevalence; and sociodemographic data (MH-SVI) obtained from government databases for 3,141 counties in the United States from 2018 to 2020. Data were analyzed using generalized additive models with and without adjustments for covariates.

**Results:**

Unadjusted models show that altitude has a comparable significant negative effect on mortality rates for Black and white populations. When including all covariates, altitude continues to have a significant protective effect against stroke fatalities in white populations (retaining 26.6 and 53.8% of the effect magnitude for cardiovascular disease (CVD) and stroke mortality, respectively), a non-significant effect in the Black population, and a detrimental effect in Hispanic and Asian Pacific populations for CVD mortality (a 21.6 and 39.1% effect increase, respectively).

**Conclusion:**

Our findings add to the growing body of evidence that environmental factors add to disparities between racial groups and play a significant role in CVD and stroke mortality. The effect of altitude is comparable in size to that of smoking, medication usage, and social determinants of health.

## Introduction

Among 369 diseases studied across 204 countries, the 2019 Global Burden of Diseases study (Vos et al., 2020) found that ischemic heart disease and strokes ranked first and second as the leading causes of increased disability-adjusted life years (DALYs), which is a metric of disease burden that represents the number of productive years lost to health issues or premature death. This report showed that stroke and ischemic heart disease ranked at the top of the list for the age groups of 50 to 74 years old and 75 years and older and ranked within the top 10 for people aged 25 to 49 years old. This suggests that strokes are not a health concern solely for the 50-plus age group. Strokes often lead to poor outcomes, with 61% of people dying or acquiring a disability within 12 months of their cerebral vascular accident (Lanas and Seron, 2021). Strokes also contribute a massive amount to healthcare costs; the estimated global cost of strokes was ~US$721 billion (Feigin et al., 2022). The World Stroke Organization states that the burden of strokes across the globe between the years 1990 and 2019 has increased, with a 70% increase in incidence, a 43% increase in deaths, and a 102% increase in prevalence (Feigin et al., 2022).

As for the United States, stroke fatalities have not significantly changed between 1999 and 2018 (12.62 to 11.81 per 100,000 people) (Song et al., 2021). However, when trends are evaluated on a per-county basis, they show geographical disparities in stroke deaths, most notoriously in the southeastern part of the country, which are mostly attributed to differences in sociodemographic and environmental factors (Howard and Howard, 2020; Song et al., 2021). A notable characteristic of the southeast region of the country is that it is situated at a low altitude; often 300 m above sea level or less. In the United States, there are close to 30 million people living at altitudes higher than 500 m and over 8 million living at altitudes above 1,500 m above sea level (Tremblay and Ainslie, 2021). Higher altitude has been shown to provide an edge in reducing poor outcomes of respiratory-related illnesses, such as COVID-19 (Bridgman et al., 2022), asthma (Hashimoto et al., 2018), and lung cancer (Simeonov and Himmelstein, 2015), as well as in cardiovascular risk factors and events (Savla et al., 2018; Faber et al., 2021; Wang et al., 2022). Higher altitude has also been shown to improve biomedical profiles such as lipid panels (Zaman et al., 2021), hemoglobin, and hematocrit levels (Villafuerte et al., 2022). When considering the relevance of altitude to respiratory and cardiac function, a 2017 study found significant geographic effects in stroke mortality (Roth et al., 2017) and a 2012 study detected some associations between ischemic heart disease and altitude; however, the effect was not consistent across all altitudes, suggesting that other effects may be confounded (Ezzati et al., 2012).

Studies examining adaptations to altitude in human and animal populations over time have led to observations that may highlight specific physiological processes that play a role in why altitude provides a benefit, as seen in Andean populations that display lower alveolar ventilation, lower pulmonary vasoconstriction responses to hypoxic scenarios, larger lung volumes, and more efficient cardiac O_2_ utilization (Julian and Moore, 2019).

This ecological cross-sectional study was designed to evaluate the effect of altitude on stroke outcomes. There is a need to further investigate the association as stroke presents a large global burden and it could result in changes to healthcare treatments that may be contingent on a geographical basis. This study can help guide future endeavors of other researchers and provide a foundation for the further examination of the health benefits, or potential drawbacks, of living at a moderate or high altitude.

## Materials and methods

### Stroke data

Stroke mortality rates (per 100,000 people) and hospitalizations data were obtained from the Interactive Atlas of Heart Disease and Stroke published by the Centers for Disease Control and Prevention's (CDC) Division for Heart Disease and Stroke Prevention (US Center for Disease Control, 2020). The Atlas pulls heart disease and stroke data from the Deaths National Vital Statistics System from the National Center for Health Statistics and hospitalization information from the Centers for Medicare and Medicaid Services, Medicare Provider Analysis and Review. This dataset contains mortality and hospitalization rates per race and age group (all ages, people over 35 years old, and hospitalizations for people over 65 years old) for cardiovascular disease (CVD), total stroke, ischemic stroke, and hemorrhagic stroke per county. Medication non-adherence in the Atlas was sourced from prescription drug claims covered by Medicare Part D for patients 65 or older within the same database. All heart disease and stroke information used in the analysis was from the most recent data available (2018–2020). All stroke data used in this study are publicly available.

### Altitude data

Altitude data per county used in this study was sourced from a study by Simeonov and Himmelstein (2015) evaluating lung cancer prevalence in association with altitude. These altitude data do not reflect the simple average as they include population dispersion adjustment that weighs the population exposure to altitude within the county. This population dispersion adjustment developed by Simeonov and Himmelstein represents census block population data variation within counties and was used to accommodate population dispersion in a more granular way since people are not uniformly distributed across a county.

### Minority health social vulnerability index

To address sociodemographic covariates, we used the Minority Health Social Vulnerability Index (MH-SVI) obtained from the US Department of Health and Human Services (HSS) MH-SVI website (US Department of Health Human Services, 2022b). The MH-SVI was developed by the HSS in conjunction with the CDC to better identify minority communities at risk of adverse health outcomes during the COVID-19 pandemic and other public health emergencies. The MH-SVI sources its data from the CDC, the US Census Bureau American Community Survey, the Department of Homeland Security, and the Institute for Health Metrics and Evaluation. All MH-SVI data are publicly available.

The MH-SVI is an extension of the CDC/Agency for Toxic Substances and Disease Registry (CDC/ATSDR) SVI (US Department of Health Human Services, 2022a). The MH-SVI ranks each census tract on social and health-related factors and groups them into six themes. Each tract receives a ranking expressed as a percentile where higher percentile rankings are indicative of greater vulnerability to adverse health outcomes. The MH-SVI includes four themes from the CDC/ATSDR SVI (socioeconomic status, household composition and disability, minority status and language, and housing and transportation) in addition to two themes specific to the MH-SVI (healthcare infrastructure and access, and medical vulnerability).

### Medication usage index

Variables measuring usage for blood pressure medications and non-adherence for blood pressure and cholesterol-lowering medications were included from the Interactive Atlas of Heart Disease and Stroke previously described. To construct a medication usage index in a similar way to the MH-SVI, we used a percentile ranking calculated on a sum of the percentile ranks for each variable. This approach follows a similar rationale as in the MH-SVI without weighing for themes.

Three counties in the state of Alaska did not include data on our dependent variables and one county in New Mexico did not include information on the SVI; therefore, these were excluded from the analyses. The final dataset included 3,141 counties in 50 states and the District of Columbia.

### Smoking data

Data relating to the age-adjusted prevalence of smoking in adults were obtained from the most recent CDC's PLACES dataset (US Center for Disease Control, 2022). Data sources for this dataset include 2020 data from the Census Bureau and the Behavioral Risk Factor Surveillance System.

### Study design and statistical analysis

**Figure 1:**
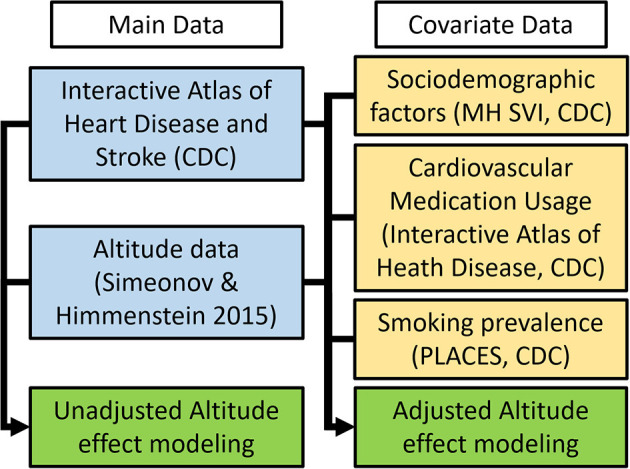
Flowchart of the study.

We designed this study as an ecological cross-sectional study that combines datasets compiled from various sources. All these sources present the data identified by county name and state; these are not ambiguous and are consistent across databases, so they were used for matching the data. Matched data were analyzed using generalized additive models (GAMs). These models provide an advantage over traditional linear models by incorporating a smoothing spline into the estimation process that can accommodate for deviations from normality in the data. Our team has used these models for similar comprehensive assessments of sociodemographic and altitude effects on COVID-19 fatalities (Bridgman et al., 2022; Gerken et al., 2022). In these models, mortality rates per 100,000 people for total CVD, total stroke, ischemic stroke, hemorrhagic stroke, and hospitalization rates were set as dependent variables. All these dependent variables were evaluated separately by race/ethnicity and by age group. The models were run without covariates (unadjusted models) and with adjustments for socioeconomic covariates using the MH-SVI (MH-SVI models) for the medication usage index (medication usage models) and for smoking prevalence (smoking models) separately, as well as with adjustments for all covariates together (smoking, MH-SVI, and medication usage models). The study strategy is summarized in Figure 1. Residual distributions were set as Gaussian. Smoothing splines were optimized iteratively starting with three degrees of freedom. All analyses were performed in SAS/STAT v.9.4 (SAS Institute Inc., Cary NC). Significant differences are presented at two thresholds: at the conventional P≤0.05 threshold and at a Bonferroni-adjusted threshold to address multiple testing burden.

Average effect sizes were calculated using the absolute values of the altitude model estimates; this was because effects could be positive or negative. To assess the contribution of covariates, these average effect sizes were compared to the unadjusted estimates and are presented as a percentage change.

## Results

**Figure 2:**
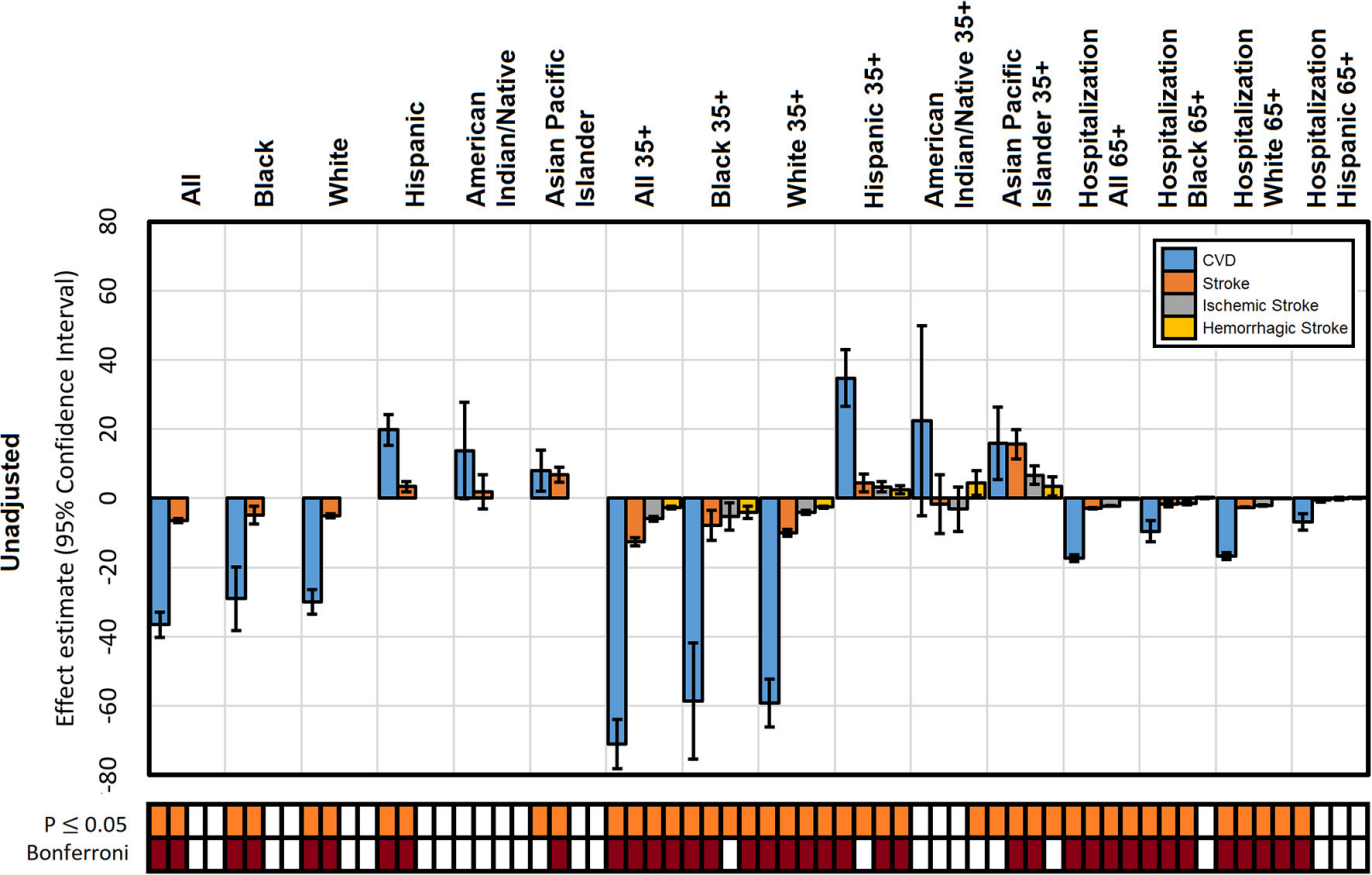
Unadjusted effect estimates of altitude on mortality from CVD, stroke, ischemic stroke, and hemorrhagic stroke and CVD hospitalization by population. The significance of each estimate is presented for a *P* ≤ 0.05 and experiment-wise Bonferroni corrected threshold.

Data from 3,141 counties were included in our analyses. Associations between altitude and mortality rates per 100,000 people for total CVD, total stroke, ischemic stroke, hemorrhagic stroke, and hospitalization rates were evaluated through GAMs that included unadjusted, sociodemographic factor adjusted, medication usage adjusted, smoking prevalence adjusted, and combined sociodemographic, medication usage, and smoking prevalence adjusted. Unadjusted model findings (Figure 2) show that altitude has a strong effect (reaching Bonferroni significance) on mortality and hospitalization rates; however, this effect displayed discrepancies across races and age groups. For mortality rates, where Black and white populations gained a protective effect, the opposite was found for the Hispanic and Asian Pacific Islander populations. It was notably that the effect was more pronounced when including only people aged 35 years or older. The effect on hospitalizations for people over 65 years of age was much smaller but also reached Bonferroni significance.

**Figure 3:**
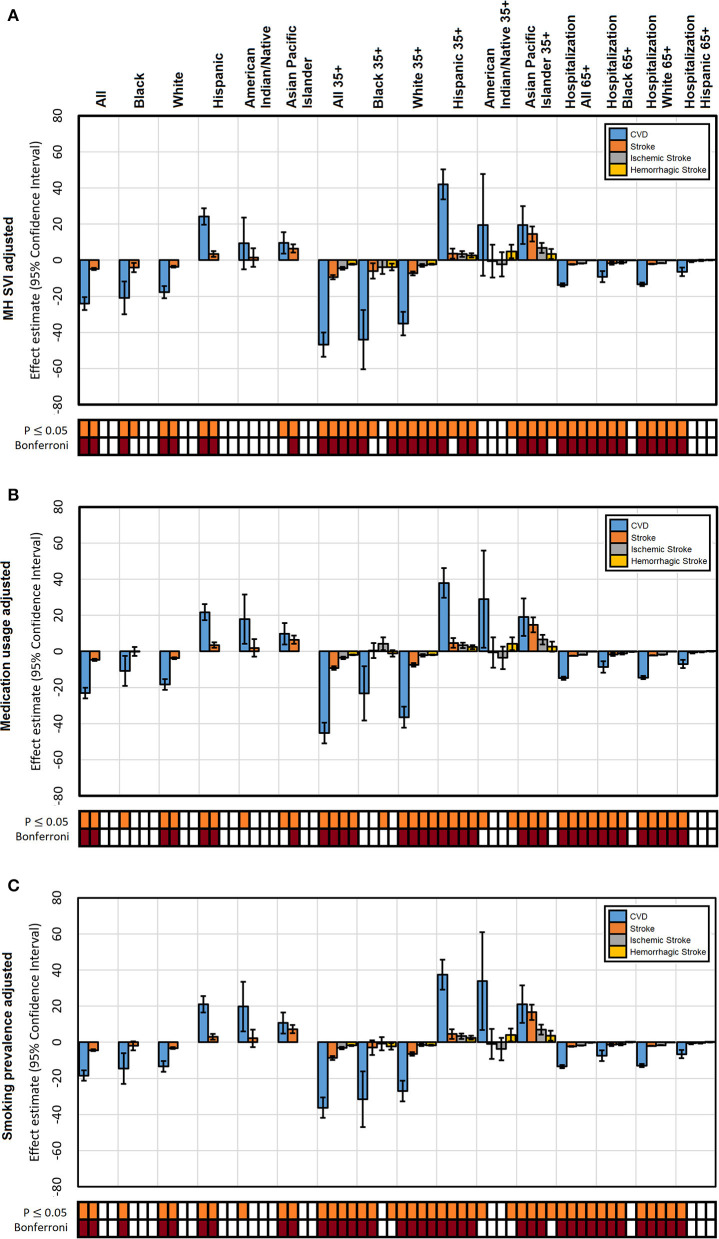
Individual factors adjusted effect estimates of altitude on mortality from CVD, stroke, ischemic stroke, and hemorrhagic stroke and CVD hospitalization by population. **(A)** Adjusted by MH-SVI; **(B)** cardiovascular medication usage; **(C)** smoking. Significance of each estimate is presented for a *P* ≤ 0.05 and experiment-wise Bonferroni corrected threshold.

When considering sociodemographic factors (Figure 3A), the protective advantage for total stroke mortality for the Black population mostly drops its Bonferroni significance, suggesting that there was a strong sociodemographic component confounding this effect. In general, the effect of altitude continues to be significant across most groups even after controlling for MH-SVI. This phenomenon was also evident when adjusting for medication usage (Figure 3B), although the addition of this confounder has a larger effect than MH-SVI in reducing the effect of altitude, again mostly in the Black population. Smoking-adjusted models show a similar pattern of reduction that was also larger than MH-SVI but similar to that of medication usage; nevertheless, the effect of altitude remains significant across most groups. The effect of altitude did not drop its Bonferroni significance in Black adults when adjusting for smoking (Figure 3C). The effect on hospitalizations over 35 years remained significant in the SVI, medication, and smoking models; and it was only slightly reduced compared to the unadjusted models.

**Figure 4:**
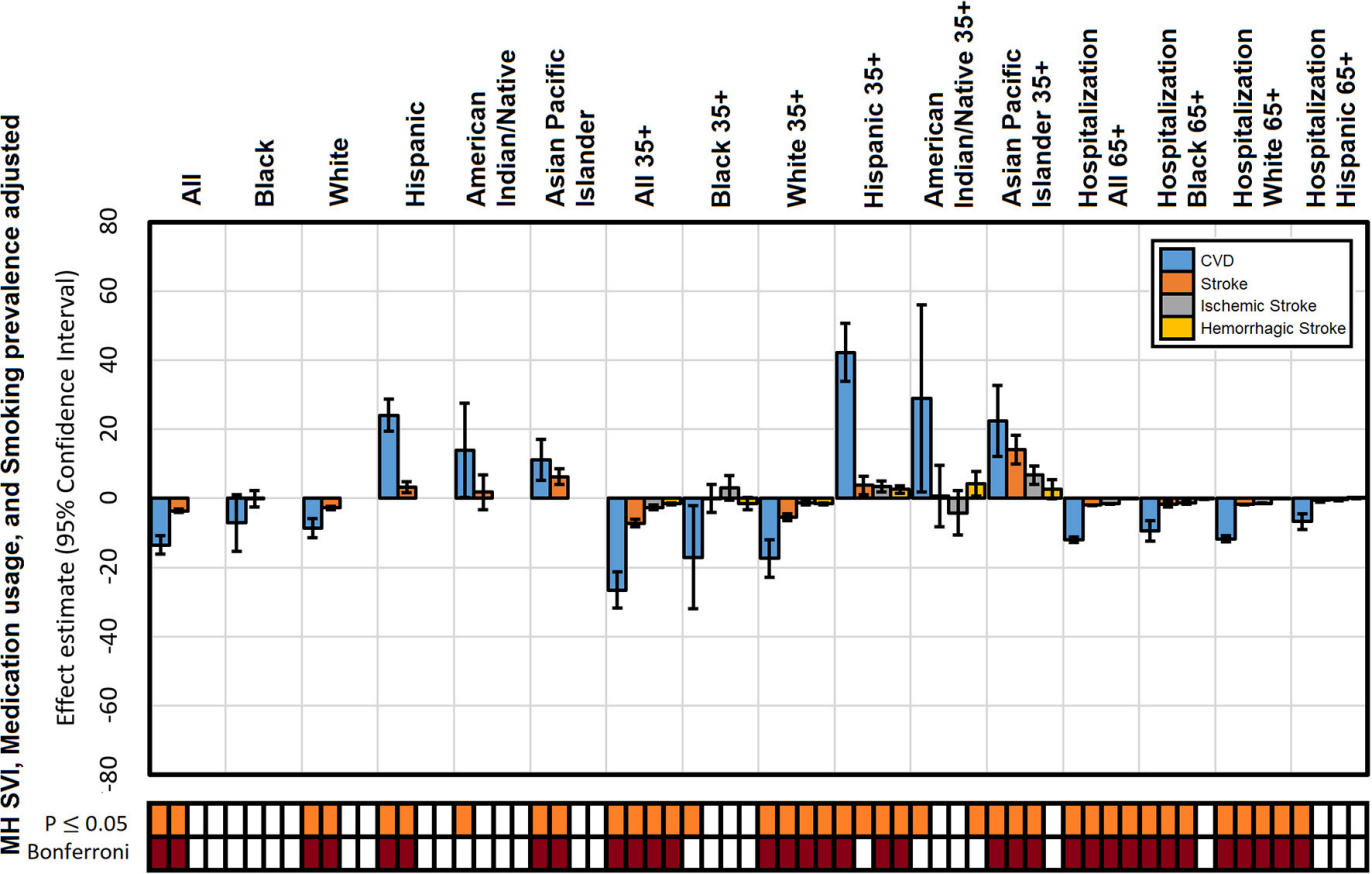
Adjusted effect estimates of altitude on mortality from CVD, stroke, ischemic stroke, and hemorrhagic stroke and CVD hospitalization by population: adjusted by MH-SVI, cardiovascular medication usage, and smoking. The significance of each estimate is presented for a *P* ≤ 0.05 and experiment-wise Bonferroni corrected threshold.

When including all covariates in the mortality models, altitude continues to display a strong effect that was consistently protective to the white population, non-significant to the Black population, and detrimental to the Hispanic and Asian Pacific Islander populations. Significance patterns for the combination of these confounder effects (Figure 4) suggest a correlation structure between them. In other words, we state that sociodemographic disparities across race groups influence medication usage among them.

Overall, the addition of covariates to the models diminished the effect of altitude (Table 1) but did not eliminate it for all groups and all types of mortality rates evaluated. The largest reduction of the altitude effect occurs in the combined covariate models with a reduction of ~38%. These findings suggest that there is a strong effect of altitude on stroke fatalities, which is expressed differentially across races and age groups. This effect remains large, ~62%, even after adjusting to sociodemographic factors and cardiovascular medication usage.

**Table 1:** Comparison of mean estimates across all models.

**Variable**	**Unadjusted**	**SVI adjusted**		**Medication usage adjusted**		**Smoking prevalence adjusted**		**All covariates adjusted**	
	**Mean Estimate**	**Mean Estimate**	**Change (%)**	**Mean Estimate**	**Change (%)**	**Mean Estimate**	**Change (%)**	**Mean Estimate**	**Change (%)**
Across All	11.38 (1.85)	9.14 (1.86)	19.7	8.54 (1.76)	25.0	8.36 (1.76)	26.5	7.08 (1.75)	37.8
CVD	28.08 (3.98)	22.19 (3.98)	21.0	21.05 (3.74)	25.0	20.34 (3.76)	27.6	17.04 (3.71)	39.3
Stroke	5.51 (1.14)	4.48 (1.17)	18.7	4.01 (1.12)	27.2	4.29 (1.12)	22.2	3.43 (1.14)	37.7
Ischemic Stroke	3.43 (0.87)	2.88 (0.89)	16.2	2.83 (0.84)	17.5	2.44 (0.84)	28.9	2.58 (0.85)	24.8
Hemorrhagic stroke	2.01 (0.52)	1.95 (0.54)	2.6	1.47 (0.52)	27.0	1.63 (0.52)	18.6	1.46 (0.52)	27.2

For this comparison, the effect of altitude for the unadjusted model is set as the reference. All covariate-adjusted models show the change of altitude effect.

## Discussion

Studies exploring the relationship between stroke and altitude have been conducted in locations outside of the United States or have only examined the effect of altitude in isolation without the simultaneous assessment of other variables (Ortiz-Prado et al., 2021, 2022). In our study, we evaluated stroke and altitude in the context of the United States on a per-county basis while controlling for confounders of sociodemographic factors, smoking prevalence, and medication usage. Overall, this data analysis shows that the effect of altitude has mixed protective and harmful effects across racial groups; however, it is important to keep in mind that these comparisons across racial groups are derived from aggregate data that is unable to isolate effects. County-level aggregate data works as a community descriptor that is specific to its own context.

Findings observed in the unadjusted models remain strong (reaching Bonferroni significance) after controlling for MH-SVI, smoking, and medication usage. One systematic review examining chronic high-altitude exposure and ischemic strokes showed that high-altitude exposure has a dose-dependent effect on the incidence and morbidity of strokes (Ortiz-Prado et al., 2021). A systematic review suggested that living at altitudes between 1,500 and 3,500 m was associated with a reduced risk of stroke, while living above 3,500 m was associated with an increased risk of stroke (Ortiz-Prado et al., 2022). Counties at an altitude of 3,500 m are rare in the United States; therefore, this effect inversion previously reported could not be assessed in these data. In addition, studies described in both reviews lack simultaneous assessments of stroke that consider altitude, race effects, and other sociodemographic factors. There are studies that show that living at higher altitudes is associated with decreased mortality from a variety of other causes. Moderate altitudes, 1000 m or less, were associated with decreased all-cause mortality from cerebrovascular diseases and cancers when compared to altitudes <250 m (Burtscher et al., 2021). The Framingham Risk Score, a sensitive prediction model used for coronary artery disease risk (Duttagupta et al., 2022), was negatively associated with altitude, especially evident in Peruvian adults living at altitudes >2,500 m (Hernández-Vásquez et al., 2022). Another study based in Switzerland found that there was a 12% decrease in risk of stroke-related death at altitude vs. at sea level (Faeh et al., 2009). Unlike previous studies performed in countries with more homogenous populations, such as Austria, Ecuador, and Peru, this study evaluates the effect of altitude on stroke in a racially heterogeneous population – the United States. A 2012 US-based study found a dose-response protective relationship between altitude and ischemic heart disease, but there was no consistent association of altitude with stroke (Ezzati et al., 2012). Despite the vast racial, ethnic, and cultural diversity of the United States, our findings are congruent with most previous studies showing that altitude has an overall protective effect on stroke mortality.

Even when the overall effect is evident, only the white population consistently sees this protective effect. Other ethnic groups see this protective effect disappear after adjustment to sociodemographic and medication usage confounders (Black population) or the effect is consistently shown to be harmful instead of protective (Hispanic and Asian Pacific Islander populations). According to a previous study Graham (2015), all ethnic groups, when compared to white populations, have a higher risk of CVD. Black adults have been found to have higher rates of myocardial infarction, chronic heart failure, and stroke risk due to a variety of risk factors when compared to white populations. Hypertension (HTN), diabetes mellitus (DM), and obesity were included in the risk factors in this study. This narrative also fits what was observed in the southern United States region known as the “Stroke Belt.” This region has an age-adjusted stroke mortality rate in excess of 25% compared to the rest of the United States (Howard and Howard, 2020). In that same region, Black adults have a 20% higher mortality rate from stroke and are 10% less likely to have controlled blood pressure than their white population counterparts (Howard and Howard, 2020). These disparities add to physiological differences among races (Gillum, 1979; Langford et al., 2020). Black individuals have higher rates of apparent treatment-resistant HTN and may utilize more home remedies compared to the white population, which may contribute to non-adherence in the southern United States (Grzywacz et al., 2006; Cuffee et al., 2020). There are other potential contributors to disparities in stroke mortality across races that have not yet been thoroughly investigated, such as depression (Jonas and Mussolino, 2000), discrimination, and the effect of inflammation (McDade et al., 2011; Howard and Howard, 2020).

Our study reveals that altitude is harmful to the Hispanic, Asian, and Pacific Islander populations. Hispanic populations are the second largest ethnic group in the United States and comprise 19% of the population. They are well represented across all altitudes in the United States, indicating that this group's increased mortality is not likely due to the geographical distribution of this population. When examining socioeconomic aspects, Hispanic populations have a 26% lower average household income than white populations with more Hispanic individuals living in poverty (US Bureau of Labor Statistics, 2021). In our study, MH-SVI was used to account for these sociodemographic differences; however, it is possible that the disparities are much larger than that for which this metric can account. Socioeconomically disadvantaged groups have repeatedly been shown to be at increased risk of several long-term diseases including DM, myocardial infarction, and stroke (Kivimäki et al., 2020). Hispanic populations overall have increased risk factors for stroke, increased numbers of strokes, and poorer outcomes from strokes compared to white populations (Marshall et al., 2015). This may be exacerbated in certain subtypes of Hispanic populations, such as those from a Caribbean background, who exhibit an increased overall incidence of HTN (Yu, 1991; Sorlie et al., 2014). Individuals of South Asian descent often have a higher atherosclerotic cardiovascular disease (Volgman et al., 2018), all of which are associated with increased risk of adverse cardiovascular outcomes and all-cause mortality (Mottillo et al., 2010). Studies have found that Asian populations have higher rates of intracerebral hemorrhagic strokes in comparison to white populations (Tsai et al., 2013; Khan et al., 2017). Specifically, Asian American populations often have more severe strokes, have higher in-hospital mortality, and receive less usage of tissue plasminogen activator (tPA) compared to patients within the white population, which could lead to increased mortality from embolic strokes (Song et al., 2019). The lack of protective effect of altitude in Asian and Pacific Islander populations in the United States may be partially confounded by geographic distribution where ~55% of the Asian and Pacific Islander populations in the United States live in California, New York, Texas, New Jersey, and Washington (Pew Research Center, 2021). Major population centers of each of these states are at lower altitudes due to their proximity to the coast. The detrimental effect associated with Asian or Pacific Islander populations may be related to fewer members of these populations living at higher altitudes rather than a physiologic change associated with this ethnic group.

When considering the Native American population, evidence indicates that they may have an overall increased risk of DM, HTN, obesity, and tobacco smoke usage (Jernigan et al., 2010). These cumulative risk factors increase the likelihood of being predisposed to strokes, incidence of comorbidities, overall poorer access to primary care areas (Schieb et al., 2014), lack of education regarding the identification of stroke symptoms (Jillella et al., 2022), and perceived discrimination (Gonzales et al., 2021). With a quarter of Native American populations living below the national poverty line, this could potentially explain the lack of protective effect from stroke when living at altitude for this population (Breathett et al., 2020).

This analysis has shown that even when accounting for risk factors and demographic variables, altitude has a protective effect in preventing hospitalizations due to stroke. This is worth noting as the spectrum of antihypertensive agents has broadened (Jackson et al., 2008), yet we still see an overall protective effect of altitude on hospitalization rates due to stroke. This aligns with an earlier study that discussed that living at altitude appears to decrease the overall risk of having a stroke and subsequently dying from it (Ortiz-Prado et al., 2022). Therefore, altitude may serve as a further protective mechanism for individuals at elevated risk of stroke despite using pharmacologic therapy. The effect of altitude observed in our study suggests that physiologic adaptations associated with living at higher altitudes are ubiquitous (Vincent et al., 1978; Brutsaert, 2016; Moore, 2017). The physiologic adaptations that occur at altitude can be divided into two phases, acute and chronic, which are differentiated based on length of exposure to hypoxic environments (Ortiz-Prado et al., 2022). These changes include anatomical adaptations, improved cardiac output and pulmonary artery pressure, improved oxygen delivery via angiogenesis, and increased diffusion capacity of the tissues (Vincent et al., 1978). These mechanisms cause an overall decrease in hypercoagulability state from baseline and establish an additional explanation for the protective effects on stroke risk for residents living at altitude.

### Limitations and future directions

We must acknowledge the limitations of our study. We used the MH-SVI to adjust for sociodemographic factors. The MH-SVI is a tool designed to identify communities in need of assistance in the event of a natural disaster or the onset of a new pandemic. The MH-SVI was designed to include demographic information and risk factors, but it certainly does not cover all factors that could increase someone's risk of stroke or CVD. Furthermore, the medication data set utilized in this study only captures information from the Medicare population, which is comprised almost exclusively of individuals who are aged 65 years and older, and does not extend to all other patients utilizing cardiovascular-indicated therapy. There are other important environmental effects that contribute to stroke outcomes, such as extreme temperature and humidity (Zhou et al., 2017; Bai et al., 2018; Lavados et al., 2018; Li et al., 2021; Wen et al., 2023), which are more likely to affect people in vulnerable socioeconomic positions. In aggregate data, the effects of environmental factors are confounded with socioeconomic factors and altitude as covariates. This is because certain elevations are more often associated with specific atmospheric conditions. Another limitation is that the characteristics of this analysis make comparison with traditional studies difficult because of the use of aggregate data as an experimental unit. On the technical side, counties across the United States are not uniformly distributed, nor does every state have the same number of counties. This may raise issues as some states may have more counties but a similar total area when compared to a state with a smaller number of counties. Future studies could use a different geographic breakdown, such as zip codes, to further evaluate these trends on a regional level and a granular assessment of medications to control medication along with treatments to address ischemic complications due to high-altitude de-adaptation such as steroids (Zelmanovich et al., 2022). Critical to future studies could be the evaluation of differing genetic profiles of those living at high altitudes and those that had strokes, the benefits to cardiovascular health on a population's longevity, and the evaluation of benefits for someone living their entire life at altitude as opposed to moving later in life. Finally, we must be clear we are presenting an ecological cross-sectional study that is observational and non-experimental in nature and does not account for time-period effects; therefore, our study cannot determine causality.

## Conclusion

Our analysis suggests that altitude has a significant protective effect on the white population; a negative effect on the Hispanic, Asian American, and American Indian populations; and no effect on the Black population when accounting for sociodemographic factors and medication usage to control hypertension. The negative effects are likely due to increased comorbidities or additional risk factors not accounted for in this study. This study lays the foundation for further exploration and could unveil the root physiological causes of the effect of altitude.

## Data availability statement

Data used in the study is available publicly from the U.S. Center for Disease Control and the U.S. Department of Health and Human Services. Curated datasets can be made available at a reasonable request to the corresponding author.

## Author contributions

JG and NH: conceptualization, data curation, investigation, and writing—review and editing. DZ: investigation and writing—original draft. IB: data curation, formal analysis, investigation, and writing—original draft. IZ: conceptualization, formal analysis, supervision, and writing—review and editing. All authors approved the final manuscript as submitted and agreed to be accountable for all aspects of the study.
